# Slug Is A Surrogate Marker of Epithelial to Mesenchymal Transition (EMT) in Head and Neck Cancer

**DOI:** 10.3390/jcm9072061

**Published:** 2020-06-30

**Authors:** T. B. Steinbichler, J. Dudas, J. Ingruber, R. Glueckert, S. Sprung, F. Fleischer, N. Cidlinsky, D. Dejaco, B. Kofler, A. I. Giotakis, I. I. Skvortsova, H. Riechelmann

**Affiliations:** 1 Department for Otorhinolaryngology, Head and Neck surgery, Medical University of Innsbruck, 6020 Innsbruck, Austria; jozsef.dudas@i-med.ac.at (J.D.); julia.ingruber@i-med.ac.at (J.I.); rudolf.glueckert@i-med.ac.at (R.G.); felix.fleischer@student.i-med.ac.at (F.F.); nataschacidlinsky@web.de (N.C.); daniel.dejaco@i-med.ac.at (D.D.); ba.kofler@tirol-kliniken.at (B.K.); aristeidis.giotakis@i-med.ac.at (A.I.G.); herbert.riechelmann@i-med.ac.at (H.R.); 2 Institute of Pathology, Neuropathology and Molecular Pathology, Medical University of Innsbruck, 6020 Innsbruck, Austria; susanne.sprung@i-med.ac.at; 3 Department of Therapeutic Radiology and Oncology, Medical University of Innsbruck; 6020 Innsbruck, Austria; ira.skvortsova@i-med.ac.at; 4 Tyrolean Cancer Research Institute, 6020 Innsbruck, Austria

**Keywords:** image cytometry, p16, p53, cancer stem cells, E-cadherin, β-catenin, EMT, head and neck cancer

## Abstract

Background: Epithelial to mesenchymal transition (EMT) promotes therapy resistance in head and neck cancer (HNC) cells. In this study, EMT was quantified in HNC tumor samples by the cellular co-localization of cytokeratin/vimentin, E-cadherin/β-catenin and by Slug expression. Methods: Tissue samples from HNC patients were stained with antibody pairs against cytokeratin/vimentin and E-cadherin/β-catenin. Epithelial–mesenchymal co-localization was quantified using immunofluorescence multichannel image cytometry. Double positivity was confirmed using confocal microscopy. Slug was semi-quantified by 2 specialists and quantified by bright field image cytometry. Results: Tumor samples of 102 patients were investigated. A loss of E-cadherin positive cells (56.9 ± 2.6% vs. 97.9 ± 1.0%; *p* < 0.0001) and E-cadherin/β-catenin double positive cells (15.4 ± 5.7% vs. 85.4 ± 1.2%; *p* < 0.0001) was observed in tumor samples. The percentage of Slug positive cells was increased in tumor samples (12.1 ± 3.6% vs. 3.2 ± 2.6%; *p* = 0.001). Ordinal Slug scores judged by two specialists closely correlated with percentage of Slug-positive cells (Spearman’s rho = 0.81; *p* < 0.001). Slug score correlated negatively with the percentage of E-cadherin positive cells (r = 0.4; *p* = 0.006), the percentage of E-cadherin/β-catenin positive cells (r = 0.5; *p* = 0.001) and positively with cytokeratin/vimentin positive cells (r = 0.4, *p* = 0.003). Conclusion: EMT can be assessed in HNC tumor probes by cytokeratin/vimentin co-expression and loss of E-cadherin/β-catenin co-expression. Slug score provides a convenient surrogate marker for EMT.

## 1. Introduction

Epithelial to mesenchymal transition (EMT) describes the phenotypic switch of epithelial cells to mesenchymal cells. It was first described in embryonic development and wound healing [1], but is also considered essential for cancer initiation, progression and metastasis. It is defined by the cellular loss of epithelial proteins, like cytokeratins, E-cadherin, or ß-catenin and the gain of mesenchymal markers like vimentin [1,2,3,4]. EMT is a transcriptionally regulated process. Its main regulators are transcription factors, which include the Zinc finger proteins SNAI1 (Snail) and SNAI2 (Slug), E-box-binding homeobox 1 and 2 (ZEB1 and ZEB2), and Twist-related protein 1 and 2 (TWIST1 and TWIST2) [5]. Slug represses the expression of epithelial genes like E-cadherin [6]. In normal epithelial cells, Slug transcription is low, and consequently E-cadherin expression is high, which allows the formation of stable, epithelial adherent junctions. During the multi-step process of EMT, the loss of E-cadherin is an early and crucial event and is especially caused by increased Slug expression [7]. E-cadherin was therefore described as the caretaker of the epithelial phenotype [1,7,8,9]. In normal epithelial cells, the intracellular domain of E-cadherin binds to ß-catenin, maintaining the spatial and functional organization of epithelial tissue. The loss of E-cadherin releases ß-catenin from its binding site, which accumulates in the cytosol, where it is rapidly ubiquitinated and degraded by proteasomes. However, released ß-catenin also translocates to the nucleus, where it induces further EMT-related signaling pathways, including the Wnt pathway [7]. These down-stream events cause the loss of epithelial cytokeratins [10], leading to alterations in cell morphology. Moreover, they induce the expression of vimentin, actually a mesenchymal protein, which is essential for the increased migratory properties of cancer cells [1,7,11]. Often EMT is not fully exerted, but occurs only partially, which leads to the co-expression of epithelial and mesenchymal markers in cancer cells [1,2,7,12].

In our previous in vitro studies, EMT increased therapy resistance in head and neck cancer (HNC) cells [13,14]. EMT also induced migration of HNC cells, expression of matrix metalloproteinases (MMPs), DNA repair, apoptosis resistance and expression of stem cell markers like carbonic anhydrase IX (CA-IX), CD44, and Nanog [13,14,15,16,17,18]. As our knowledge of EMT is largely based on cell culture experiments, the clinical relevance of EMT in cancer has been questioned [19]. In this study, EMT was quantified in tumor samples from HNC patients using image cytometry and confocal microscopy of immunolabelled cells. We used the cellular vimentin, cytokeratin co-localization as marker for EMT [8,20]. Moreover, loss of E-cadherin, ß-catenin, and E-cadherin/ß-catenin co-expression were assessed as earlier EMT related events [8,20,21]. The expression of the EMT-related transcription factor Slug was quantified using enzyme immunohistochemical image cytometry. Additionally, semiquantitative scores of Slug immunohistochemistry were estimated by 2 experienced investigators. These scores were correlated with the quantitative results of image cytometry to determine whether Slug scoring could serve as an EMT surrogate marker in routine clinical practice. Semiquantitative Slug scores were correlated with the expression of other biomarkers including p16, Ki-67, carbonic anhydrase IX (CA-IX), CD44, survivin, excision repair cross complementation group 1 (ERCC1), matrix metalloproteinase 9 (MMP9), and programmed cell death 1 ligand 1 (PD-L1). 

## 2. Experimental Section

### 2.1. Patients and FFPE Samples

For this observational clinical study with archived human biological material, patients with incident head and neck squamous cell carcinoma (HNC) treated between 2008 and 2019 at the Department of Otorhinolaryngology-Head and Neck Surgery, Medical University of Innsbruck, were eligible. Before treatment, tumor samples were obtained during diagnostic panendoscopy under general anesthesia. Control mucosa was obtained from patients who underwent uvulopalatopharyngoplasty (UPPP), a routine surgical procedure for the treatment of snoring and obstructive sleep apnea. All tissue samples were formalin fixed and paraffin embedded (FFPE) and sectioned in 5 µm thick slices. FFPE blocks were archived (Ethics Committee of the Medical University of Innsbruck, received 10.06.2009, reference number UN3678 278/4.15) and all relevant clinical data were stored in a clinical tumor registry. Written informed consent was obtained from all included patients. This study on archived human material was approved by the Ethics Committee of the Medical University of Innsbruck (reference number: UN4535 307/4.6, received 18 November 2011).

### 2.2. Routine Immunohistochemistry and Subsample for EMT Analysis

Enzyme immunohistochemistry of tumor samples was routinely performed to aid in clinical decision-making. In 2016, Slug was added to the panel of routinely performed tumor biomarkers (Supplementary Table S1). Immunohistochemistry was completed by an universal secondary antibody (Roche Ventana, Oro Valley, AZ, USA) and the Ventana DAB Map kit (Supplementary Table S1). Biomarker expression in tumor cell areas was scored from 0 (no staining) 1 (weak staining), 2 (intermediate staining) and 3 (strong staining) according to criteria provided in Supplementary Table S2. The EMT-related transcription factor Slug was scored into 0 (0–5%); 1 (6–33%); 2 (34–66%); and 3 (67–100%) [22]. From the patients with routine immunohistochemistry for Slug, a subsample was drawn for detailed analysis of the association of Slug expression and biomarkers of EMT. This subsample included patients of whom sufficient archived FFPE samples were available. In a first step, the Slug scores in routine FFPE specimens were counterchecked by 2 investigators who agreed on an ordinal score. If their judgements were discordant a coherent judgement had to be made by inspecting the samples together. Moreover, Slug score was dichotomized as negative (scores 0 and 1) or positive (scores 2 and 3).

### 2.3. Analysis of Slug and EMT Marker Expression

#### 2.3.1. Slug Quantification Using Enzyme Immunohistochemistry and Image Cytometry

In a second step, the percentage of Slug positive cells was quantified using enzyme immunohistochemical image cytometry. The immunostained samples were counterstained with hematoxylin and acquired with a 40× magnification dry lens coupled onto a Zeiss Axio Imager Z2 Microscope (Jena, Germany) and an 8 slide automatic stage (Märzenhäuser, Wetzlar, Germany) using the TissueFAXS Plus brightfield system (TissueGnostics, Vienna, Austria). Regions in tumor cell areas without obvious artefacts were selected and analyzed using the HistoQuest (TissueGnostics) imaging analysis software. It uses algorithms for automated single-cell identification based on the blue color of hematoxylin stained nuclei. Moreover, it counts positive cells for marker-coupled DAB staining. The software creates scatterplots that allow visualizing positive and negative cells by means of a backward gating function [23]. Cut-offs for enzyme immunohistochemistry products were set on basis of negative controls. Negative controls were acquired by alternating the primary antibodies with reaction buffer or by substituting them with isotype matching immunoglobulins. These negative controls never yielded any immunostaining.

#### 2.3.2. EMT Quantification Using Immunofluorescence Multichannel Image Cytometry 

Indirect immunofluorescence staining for vimentin, cytokeratin, E-cadherin, and ß-catenin was performed using primary antibodies and secondary antibodies as described in Supplementary Table S3. For isotype controls, isotype matching mouse IgG1 and rabbit IgG antibodies were used (Supplementary Table S3). DAPI was purchased from Molecular Probes Inc., Eugene, OR, USA. Autofluorescence was reduced with the TrueView^TM^ autofluorescence quenching kit (Vector Laboratories, Burlingame, CA, USA). Fluorescence intensities were obtained on 2^14^ bit grey scale using a PCO pixelfly CCD camera (PCO AG, Kelheim, Germany) and the TissueQuest image acquisition software (TissuGnostics, Vienna, Austria). Tumor specimens were separated by investigator’s judgment into tumor cell areas and tumor stroma. In controls, specimens were divided in the epithelial layer and the lamina propria mucosae (Supplementary Figure S1). Image analysis was conducted with StrataQuest^®^ 6.0 software (TissuGnostics, Vienna, Austria). DAPI was used for single-cell identification. Cut-offs were defined using isotype controls (Supplementary Figure S2). The percentage ratio of positive cells for a specific antibody was calculated as the number of cells that showed higher fluorescence intensity than the isotype threshold divided by the total cell number. Co-localization of 2 antibodies was assumed, if fluorescence intensities of both were higher than the isotype threshold in a single cell. For comparison of HNC specimens with UPPP-specimens (controls), cellular co-localization data were not available. An intensity weighted index of the percentage of positive cells served as a measure of expression [24]. The ratio of vimentin index/cytokeratin index was used to quantify EMT [20,25,26].

#### 2.3.3. Confocal Microscopy

Double positive reaction of cytokeratin/vimentin or E-cadherin/β-catenin was confirmed using confocal microscopy. The immunostained sections were digitalized at 20× magnification utilizing a TissueFaxs Plus System coupled onto a Zeiss^®^ Axio Imager Z2 Microscope (Jena, Germany). Annotated cell borders were identified with the transmission channel.

### 2.4. Data Analysis

The total number of evaluated cells, the percentage of positive cells in in a sample and other cytometry parameters were taken from the image cytometry program. Outcome parameters of image cytometry were essentially log-normally distributed. Mean values and standard deviations were calculated on a logarithmic scale and the results were transformed back to the original scale to improve readability. Patient and control samples were compared using independent samples t-test with Satterthwaite approximation for the degrees of freedom using logarithmic data. For correlations of quantitative Slug expression and EMT biomarkers, Pearson’s r was calculated. For semi-quantitative Slug scores, correlations were performed using Spearman’s rho. Statistical analyses were performed using SPSS 26 (IBM Corporation, Armonk, NY, USA). 

## 3. Results

### 3.1. Study Population

Between 2008 and 2019, 1115 patients with incident HNC were treated at the Department of Otorhinolaryngology-Head and Neck Surgery, Medical University of Innsbruck. Routine immunohistochemistry, including Slug, was available in 355 patients. Of these, 102 patients with a sufficient amount of archived FFPE samples were chosen for a detailed image cytometric analysis of Slug and EMT markers. Oropharyngeal mucosa from healthy individuals obtained during UPPP was obtained in 12 patients. In the subset of 102 patients, most patients were male (85/102, 83%). The mean age was 63 (±12) years (Table 1). The sample subset for detailed analysis differed in 2 aspects from the total HNC population of 1115 patients treated during the observation period: T3 and T4 tumors (*p* < 0.01) and oropharyngeal tumors (*p* < 0.01) were more common than in the total HNC population. This is likely due to the amount of available FFPE samples in advanced primary and in oropharyngeal tumors. No significant differences between the subsample and the total HNC patient population were observed for p16 positivity (*p* = 0.51), gender (*p* = 0.2), age at first diagnosis (*p* = 0.55), ASA score as a measure of general health condition (*p* = 0.83) [27], histopathology (*p* = 0.11), overall survival (*p* = 0.08), and first line treatment (*p* = 0.14).

### 3.2. Routine Immunohistochemistry

**Figure 1:**
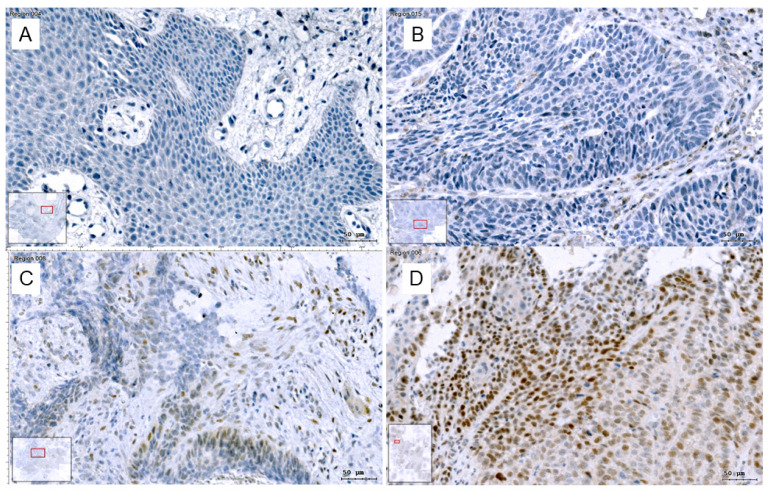
Slug IHC: Slug, immunohistochemical reaction in HNC was categorized and scored into (**A**): absent (0), (**B**): scattered weak reaction (1+), (**C**): focal strong reaction (2+) and (**D**): generalized strong reaction (3+). Images were taken in the TissueFaxs system, bars: 50 µm. With this classification, Slug positive cells in the tumor stroma are not counted.

Slug immunohistochemistry (Figure 1) was categorized as positive in 89/355 patients (25%). The correlations of Slug scores and other routine IHC scores were analyzed using Spearman’s correlation coefficient. Slug scores correlated positively with the scores for Ki-67 proliferation index (rho = 0.15; *p* = 0.005; *n* = 354), stem cell markers CAIX (rho = 0.29; *p* < 0.001; *n* = 175) and CD44 (rho = 0.18; *p* = 0.02; *n* = 160; Table 2), but not with PD-L1 score. Slug score correlated negatively with p16 positivity (rho = −0.133; *p* = 0.012; *n* = 354). In the subsample chosen for detailed analysis, 39/102 (38%) patients were rated as Slug positive by 2 investigators. In 2 samples, Slug scoring was initially discordant, and a coherent judgement was reached by inspecting the samples together.

### 3.3. Analysis of Slug and EMT Marker Expression

In general, tumor cell areas were clearly distinguishable from tumor stroma (Supplementary Figure S1). Only tumor cell areas within the tumor probes were evaluated. Evaluated tumor cell areas measured 5.7 ± 7.1 mm^2^ (mean ± SD) and contained 33,365 ± 40,335 cells. The 5 quantitative outcome parameters of image cytometry analysis were percentage of Slug positive cells using brightfield image cytometry, percentage of E-cadherin positive cells, percentage of ß-catenin positive cells, percentage of E-cadherin/ß-catenin double positive cells and percentage cytokeratin/vimentin double positive cells. These 5 parameters were essentially log-normally distributed. Mean values and standard deviations were calculated on a logarithmic scale and the values were transformed back to the original scale to improve readability. 

#### 3.3.1. Slug Quantification Using Enzyme Immunohistochemistry and Image Cytometry 

Compared to controls, Slug was upregulated in HNC samples (*p* = 0.001; Table 3). Slug scores from routine immunohistochemistry correlated closely with the percentage of Slug positive cells quantified by image cytometry (Spearman’s rho 0.81, *p* < 0.001) supporting the validity of the 2 investigators’ judgement. Moreover, it seemed reasonable to dichotomize Slug scores into negative (no and weak expression) and positive (intermediate and high expression) using a cut off at 10% Slug-positive tumor cells (Table 4).

#### 3.3.2. Gain of Cytokeratin/Vimentin Double Positive Cells

**Figure 2:**
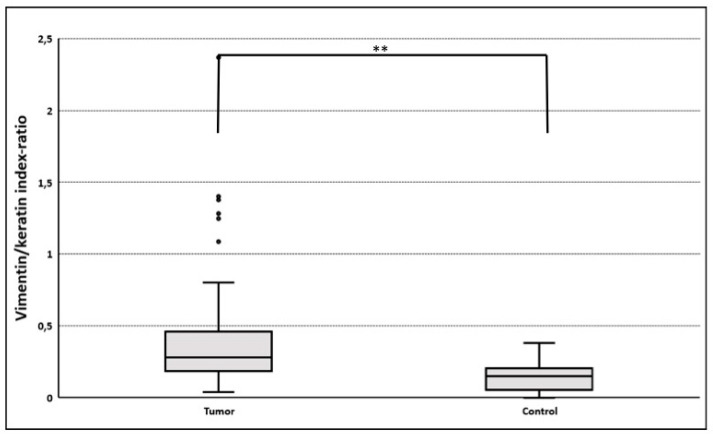
Vimentin/cytokeratin index ratio in tumor and control samples. Co-expression data based on single cell analyses were not available for controls. Instead, the ratio vimentin fluorescence index/cytokeratin fluorescence index in tumor cell areas (*n* = 74) and epithelial layer of controls (*n* = 9) was used to quantify epithelial to mesenchymal transition (EMT) [20,25,26]. A higher vimentin/cytokeratin index ratio suggests a more mesenchymal phenotype. Because of the low number of controls (*n* = 9) parametric, statistical evaluation was not possible. The median vimentin/cytokeratin index ratio was 0.28 (LQ 0.19; UQ 0.46) in tumor cell areas and 0.15 (LQ0.05; UQ 0.21; ** *p* < 0.005).

**Figure 3:**
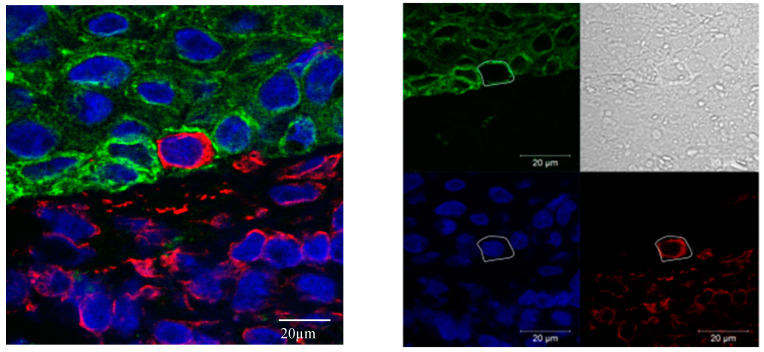
Cellular co-localization of vimentin in cytokeratin in confocal microscopy. Co-localization of mesenchymal vimentin (**red**) and epithelial pan-cytokeratin (**green**) in HNC in confocal immunofluorescence microscopy (**left side**). Single channel, annotated cell border identified with the transmission channel (**right side**, scale 20 μm). Cell nuclei are stained blue. Pictures were acquired in TissueFax Sytem.

**Figure 4:**
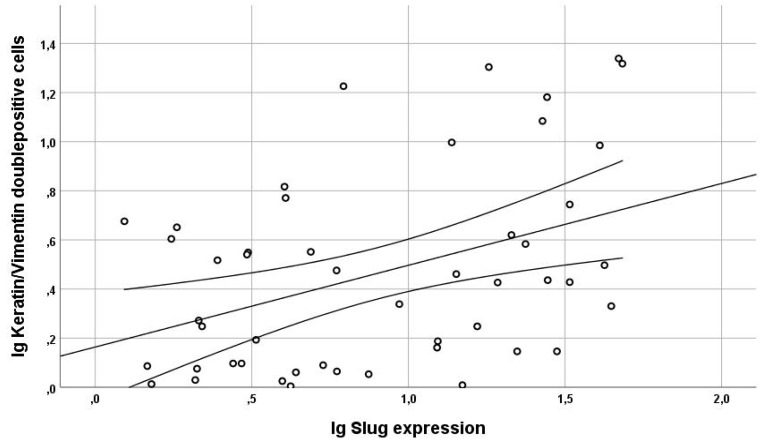
Correlation Slug expression and cytokeratin/vimentin co-expression. Regression analysis of the logarithmic percentage of Slug positive cells with the logarithmic percentage of cytokeratin/vimentin double-positive partial EMT cells. The two curved lines indicate the 95%-confidence interval, whereas the straight line displays the regression line. The percentage of Slug-positive cells in the tumor cell areas correlated positively with the percentage of cytokeratin/vimentin double-positive partial EMT cells (r = 0.41; R^2^ = 0.17; *p* = 0.005).

**Figure 5:**
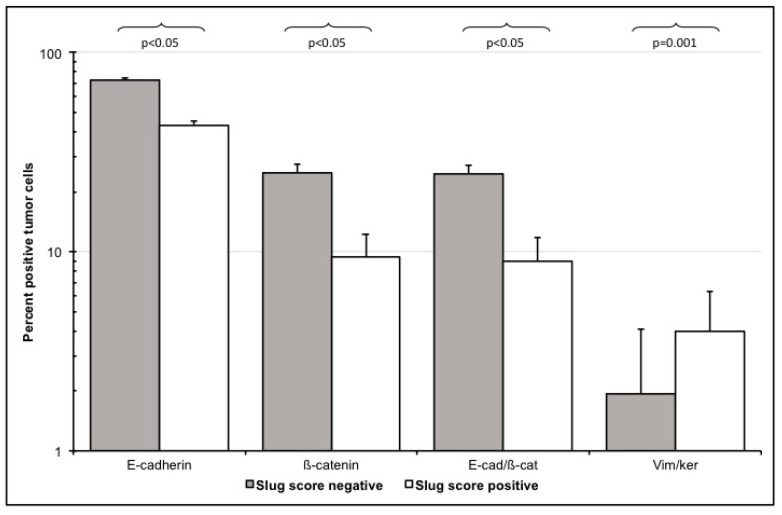
EMT marker expression in Slug-score positive and Slug-score negative patients. HNC specimens of 72 patients were semi-quantitatively scored based on Slug immunohistochemistry by 2 investigators and then dichotomized in Slug positive and Slug negative (*x*-axis). Using multichannel immunofluorescence image cytometry, expression of EMT related proteins in tumor cells were quantified as percentage of positive cells (*y*-axis). Slug positive HNC specimens had a lower expression of epithelial E-cadherin (*p* < 0.05) and ß-catenin (*p* < 0.05), a lower E-cadherin/ß-catenin co-expression (*p* < 0.05) and a higher cytokeratin/vimentin co-expression (*p* = 0.001) indicating simultaneous downregulation of epithelial and upregulation of mesenchymal proteins consistent with EMT.

EMT upregulation in HNC tumor tissues was best quantified using vimentin/cytokeratin index ratio. A high index ratio is an indicator of vimentin overexpression and cytokeratin loss, which is characteristic for EMT [20,25,26]. In tumor cell areas, the median vimentin/cytokeratin index ratio was almost twice as high (0.28) as in the epithelial layer of oropharyngeal mucosa of control patients (0.15; *p* < 0.005; Figure 2). The simultaneous occurrence of the epithelial protein cytokeratin and the mesenchymal protein vimentin in a single cell of epithelial origin is a biologically intuitively proof of partial EMT. Therefore, these vimentin/cytokeratin double positive cells served as a reference for partial EMT in this study [28]. The actual cellular co-localization of vimentin and cytokeratin in HNC tumor cells observed in image cytometry was verified by confocal microscopy (Figure 3). The percentage of Slug-positive cells in tumor cell areas correlated positively with the percentage of cytokeratin/vimentin double-positive cells (r = 0.41; R^2^ = 0.17; *p* = 0.005; Figure 4). The percentage of cytokeratin/vimentin double-positive cells was 2.65 ± 2.35% in the tumor cell areas. In Slug-positive tumors, 4.0 ± 2.6% of tumor cells were cytokeratin/vimentin double positive compared to 1.9 ± 1.8% in Slug negative tumors (*p* = 0.001; Figure 5). 

#### 3.3.3. E-Cadherin and ß-Catenin Single Positive Cells 

Loss of E-cadherin expression is considered an early event in EMT in HNC [1,7,9]. E-cadherin expression was significantly downregulated in HNC tumor cell areas, where it was 56.9 ± 2.6% (Table 3). The percentage of Slug-positive cells (Table 3) correlated negatively with the percentage of E-cadherin positive cells (Pearson’s r = 0.4; R^2^ = 0.16; *p* = 0.006). However, loss of E-cadherin alone did not significantly correlate with vimentin/cytokeratin co-expression (Pearson’s r = −0.144, *p* = 0.3).

Beta-catenin expression was substantially downregulated in HNC (Table 3). In tumor cell areas, mean percentage of ß-catenin positive cells was 15.4 ± 5.7%. It correlated negatively with the percentage of vimentin/cytokeratin double positive cells (Pearson’s r = −0.353; *p* = 0.009). Beta-catenin was mainly localized membranous in the HNC samples, a nuclear localization was not observed.

#### 3.3.4. Loss of E-Cadherin/Beta-Catenin Double Positive Cells

E-cadherin/ß-catenin double positive cells represent a stable state of epithelial cells within epithelial tissues [29,30]. While there were high numbers of E-cadherin/ß-catenin co-expressing cells in the epithelial layer of normal oropharyngeal mucosa specimens (85.4 ± 1.2%), these cells were infrequent in tumor cell areas of HNC patients (15.4 ± 5.7%; *p* < 0.0001; Table 3). The percentage of Slug positive cells in tumor cell areas correlated with the loss of E-cadherin/ß-catenin double-positive cells (Pearson’s r = 0.5; R^2^ = 0.25; *p* = 0.001). Moreover, loss of E-cadherin/ß-catenin double positive cells correlated with the percentage of vimentin/cytokeratin double-positive cells (Pearson’s r = −0.344; *p* = 0.01).

## 4. Discussion

In previous in vitro studies in HNC cells we observed that EMT is of high relevance in tumor progression and therapy resistance [13,14,15,16,17,18]. However, the clinical relevance of EMT in cancer has been questioned as EMT is difficult to measure in tumor samples and EMT is frequently not observed in histopathological examinations of cancer tissues by pathologists [31,32]. As a consequence, surrogate markers for EMT, e.g., loss of E-cadherin expression or increased vimentin expression were used in cancer tissue [19,33,34]. However, loss of E-cadherin can also be caused by other events than EMT, e.g., disruptive mutations [6]. Dependence on individual EMT marker genes has limited the ability to reliably identify and characterize EMT in human tumors, so the relevance of EMT for tumor progression in vivo is still not proven [35].

To address these challenges, we questioned whether EMT can be detected and quantified in HNC tissues. We also questioned if there is a positive correlation between EMT-typical cellular protein expression and the expression of the EMT related transcription factor Slug. Control tissues from UPPP patients served to support the validity of the used methods. However, a systematic case-control comparison of EMT markers in HNC patients and controls was not performed.

### 4.1. Methods

EMT was identified by loss of epithelial proteins including cytokeratin, E-cadherin, and ß-catenin, increase of the mesenchymal protein vimentin, and the expression of the EMT-related transcription factor Slug in tumor cells of HNC patients. These parameters were considered established and biologically sound biomarkers for EMT [1,2,5,6,7,8,9]. The investigations focused on tumor cell areas with a high proportion of epithelial cells [8]. We thought that EMT in the tumor stroma might be overestimated, since epithelial cells that have left their normal epithelial environment as a result of the EMT can be found there preferentially. In addition, the restriction to tumor cell areas made the comparison with the epithelial layer of normal oropharyngeal mucosa more plausible. 

Multichannel immunofluorescence microscopy served to quantify EMT-related biomarkers and intracellular co-localization [15,23]. Brightfield immunocytometry was used for quantification of the EMT related transcription factor Slug. The basis of these methods is that fluorescence or color intensity correlate with protein expression [24]. Various quality assurance measures had been taken to confirm reliability of this correlation. These include titration tests for antibody concentrations and exposure times, suppression of autofluorescence and background correction. The results of image cytometry studies depend on manually adjustable parameters. A parameter with great influence on the results is the intensity threshold for the assignment as a positive or negative cell. This threshold was set using isotype controls (Supplementary Figure S2). The outcome was the percentage of positive cell count/total cell count and an intensity index taking also the fluorescence intensity of positive cells into account [20,25,26]. Due to series of experiments we consider the image cytometry results trustworthy. In contrast to flow cytometry, partially sectioned cells pose a problem with image cytometry. Due to thin tissue slices (5 µm) and the selection of a minimum nucleus area of 40 µm^2^, peripherally sectioned cells were largely excluded. However, cytoplasmic overlays can occur, which pretend cellular co-localization. Using an interactive forward and backward connection between histogram and scattergram data on the one hand and original and shaded images on the other hand, a direct comparison of processed data and the acquired image was possible. This visualization supported that co-localizations claimed by StrataQuest software were actually true co-localizations. Moreover, this was in good agreement with the results of confocal microscopy examinations, which is the standard method to detect cellular co-localization (Figure 3).

### 4.2. Slug Expression and EMT in HNC

When compared with normal oropharyngeal mucosa, the EMT transcription factor Slug was overexpressed in HNC tumor cells (*p* = 0.001). Slug expression was almost 4-fold higher in HNC. This is in line with other reports [22]. Slug overexpression was associated with EMT-typical changes of epithelial and mesenchymal markers in tumor cells. It significantly correlated with downregulation of the epithelial markers E-cadherin and ß-catenin and both, E-cadherin and β-catenin in tumor cells (*p* < 0.005; Figure 5). Loss of E-cadherin and β-catenin are considered indicators of an early stage of EMT [6,7,8,9]. Particularly E-cadherin/β-catenin double-positive cells, which represent a stable epithelial phenotype in normal epithelium, were nearly five-fold lower in HNC tumor cell areas (*p* < 0.001). Especially, membranous localization of β-catenin was reduced in the tumor samples. Membranous expression of β-catenin exerts a restrictive effect on tumor cell movement and growth. Loss of β-catenin expression on the cell surface is an EMT associated event and increases cell motility, growth, and transformation and thus promote tumorigenesis [36,37,38].

Cytokeratin/vimentin co-expression in tumor cells indicates partial EMT [28,39,40]. Almost 3% of HNC tumor cells were cytokeratin/vimentin double positive. This was also confirmed with confocal microscopy (Figure 3). The percentage ratio of cytokeratin/vimentin double-positive cells significantly correlated with Slug expression (r = 0.41; *p* = 0.005; Figure 4). In a recently published study by Puram and colleagues single cell RNA-sequences were used to analyze EMT in HNC tissues and identified an EMT-like expression program. This program included several classical EMT markers (like podoplanin, vimentin, and integrin alpha-5), extracellular matrix genes (matrix metalloproteinases, integrins, and laminins), and Transforming Growth Factor Beta Induced (TGFBI) [35]. They suggested that partial EMT, where cells remain cytokeratin positive, occurs in HNC tissue by increased Slug expression, whereas other EMT-related transcription factors like Twist1/2 and SNAI1 might cause “full” EMT [35]. This link between Slug and partial EMT was demonstrated before in embryonic development, especially in renal tubulogenesis [41] and cutaneous wound re-epithelialization, where partial EMT is associated with collective migration of epithelial cells allowing wound closure [42]. In cancer, partial EMT associated with Slug expression, might be of special relevance for collective migration of cancer cells facilitating invasion, intravasation and subsequent metastasis [39].

Although the correlation of Slug expression with the investigated EMT-related biomarkers was highly significant, this correlation was not very strong. Only about 20% of the variation in EMT biomarkers observed in this study could be explained by Slug expression. Slug is actually just one of several EMT-related transcription factors that likewise contribute to the observed EMT phenotypes in HNC samples [5,43]. In summary, we consider our observations on Slug- and EMT-related biomarkers in HNC to be biologically plausible and consistent with our current knowledge of EMT-related mechanisms [1,7,8,9]. Our data suggest that EMT is not only an artificial phenomenon under cell culture conditions, but is a frequent event in head and neck cancer patients, which is at least partially regulated by the transcription factor Slug.

### 4.3. Slug Scores as A Clinical Surrogate Marker for EMT

Multi-channel immunofluorescence cytometry and immunohistochemical image cytometry are methodologically complex, costly, and time-consuming. This makes their use in routine diagnostics of HNC tissue specimens difficult. We questioned if semi-quantitative scores of the expression of the EMT-related related transcription factor Slug may serve as a surrogate to the laborious quantification of EMT using image cytometry. The percentage of Slug positive cells as measured with image cytometry correlated closely with an easy to evaluate and reproducible Slug score (Spearman’s rho = 0.81; *p* < 0.001). The Slug score was judged by two investigators on an ordinal scale in 102 patients with only 2 discordant results. This score was based on a previously published score for HNC [22]; however, quantitative results revealed that actual cell counts were somewhat lower (Table 4). Based on quantitative data of Slug expression it became obvious that the ordinal scores can be dichotomized into Slug positive and negative specimens at a cut off of 10% positive tumor cells. Slug positive tumors had a significantly lower expression of epithelial proteins E-cadherin and ß-catenin, a lower E-cadherin/ß-catenin co-expression and a higher vimentin/cytokeratin co-expression indicating simultaneous upregulation of mesenchymal proteins consistent with partial EMT (Figure 4 and Figure 5). As an alternative to estimating the percentage of Slug positive cells, Slug expression can also be divided into absent (0), scattered weak reaction (1+), focal strong reaction (2+), and generalized strong reaction (3; Figure 1). This scoring is easier to estimate and appears more realistic for the authors than the scoring suggested previously [22].

Ordinal slug scores were recorded in a larger cohort of more than 300 patients and correlated with other EMT-related tumor markers (Table 2). Biologically, the observed correlations support that Slug scores can serve as surrogate markers for EMT. The relationship between EMT and the development of cancer stem cells has been extensively investigated. In line with this, we observed a correlation between Slug scores and the expression of the stem cell-related proteins CA-IX, CD44, and MMP9 [28,44,45,46,47]. Slug scores correlated with survivin and ERCC1 expression, which are both related to therapy resistance [43,44].

Furthermore, the review of the IHC probes by a pathologist revealed that higher Slug scores correlated with higher pathological grades. The relationship between high pathological grading and EMT was already described for other EMT-related signaling factors, like Secreted Protein Acidic and Rich in Cysteine (SPARC) and TEA domain TF 4 (TEAD4), in HNC [48,49] and might be a consequence of increasing de-differentiation of the cancer cells as a consequence of EMT [44]. Furthermore, Slug positive cells were enriched in the invasion front of the cancer biopsies, highlighting the role of EMT in tumor invasiveness [13,44].

The inverse correlation between Slug and p16 positivity, as a marker for human papilloma virus (HPV) infection, requires adjustment for p16 effects when examining the clinical effects of Slug expression (*p* = 0.012). There are several studies in HPV positive cervical cancer cells (especially HeLa cells) that report that the HPV related oncogenes E6 and E7 might repress E-cadherin expression independent of Slug and consequently induce EMT in cervical cancer [50,51,52,53,54]. In HNC the association between HPV status and EMT is even less known, but the results also support a positive association between HPV status and EMT in vitro [55]. However, as HPV induced E-cadherin repression and subsequent EMT are described as Slug independent and might be caused by increased histone methylation [54], these results might not be contradictory per se. 

### 4.4. Limitations

In general, retrospective studies with archived human tissues are subject to a selection bias because sufficient sample material is readily available for large primary tumors that are easily accessible. This is the likely reason why advanced primary tumors and oropharyngeal primaries were overrepresented in this study. However, these two factors did not confound the EMT-related parameters examined in this study. Another possible bias in this study was that Slug was included in our routine immunohistochemical panel in 2016 and samples from some previously treated patients were subsequently stained. A possible source of observer bias was the separation of specimens in tumor cell areas and stroma or epithelial layer and lamina propria, but EMT-related biomarkers in tumor cell areas or tumor stroma did not differ substantially. 

## 5. Conclusions

In tumor samples of patients with HNC, EMT can be quantified using multichannel fluorescence image cytometry of epithelial and mesenchymal proteins. EMT marker correlate with the expression of the EMT-related transcription factor Slug. Semi-quantitative scoring of Slug expression is a useful surrogate marker for EMT in HNC. 

## Figures and Tables

**Table 1:** Study population. Clinical features of 102 patients with head and neck cancer who agreed to submit a histological sample for this study.

Variable	Value	Count	Percent
Sex	male	85	83%
	female	17	17%
Age	≤50	15	15%
	51–60	34	33%
	61–70	32	31%
	71–80	14	14%
	>80	7	7%
Tumor site	lips/oral cavity	10	10%
	oropharynx	49	48%
	hypopharynx	19	19%
	Larynx	19	19%
	other	5	5%
UICC stage	stage 1	10	10%
	stage 2	10	10%
	stage 3	20	20%
	stage 4a	49	48%
	stage 4b	6	6%
	stage 4c	7	7%
P16 status	negative (<60%)	74	73%
	positive (≥60%)	28	27%

**Table 2:** Correlation of Slug scores and various biomarker scores obtained with routine immunohistochemistry in tumor specimens of head and neck cancer (HNC) patients.

Marker	Number of Available Samples *	Spearman’s Rho	*p*-Value
Ki67	354	0.148	0.005
p16	354	−0.133	0.012
CA-IX	175	0.285	<0.001
CD44	160	0.176	0.026
Survivin	138	0.259	0.002
ERCC1	117	0.262	0.004
MMP9	352	0.190	<0.001

* The number of available samples is highly variable due to modifications in our routine immunohistochemistry staining procedure over the last years, which was continuously adapted to reflect recent scientific data.

**Table 3:** Percentage of EMT marker positive cells quantitatively measured by image cytometry in tumor and control samples.

EMT Marker	Tumor		Control		Sig.
	Mean ± SD (%)	*n*=	Mean ± SD (%)	*n*=	
Slug	12.1 ± 3.6	61	3.2 ± 2.5	11	0.001
E-cadherin	56.9 ± 2.6	64	97.9 ± 1	12	0.049
Beta-catenin	15.4 ± 5.7	64	86.2 ± 1.2	12	0.001
E-cadherin/ß-catenin doublepos.	15.4 ± 5.7	64	85.4 ± 1.2	12	0.001

**Table 4:** Results of Slug scores and quantification using image cytometry. Cross table of the estimated Slug expression on an ordinal scale based on the classification of Cappellesso and colleagues [22] and the results of the Slug quantification using image cytometry. A modification of the percentage ranges of the original classification according to the right column of the table is proposed. Slug was scored into absent (0; 0%), scattered weak reaction (1; ≤10%), focal strong reaction (2; ≤30%) and generalized strong reaction (3; >30%). This data suggests dichotomization in no and weak to negative (*n* = 30) and intermediate and strong to positive (*n* = 30) Slug expression, i.e., positive if >10% of tumor cells are Slug positive.

Slug IHC ^1^	Number of Patients	Image Cytometry ^2^	Modified Ranges ^3^
0 (no)	21	3.7 ± 2.3	0%
1 (weak)	9	5.6 ± 1.6	≤10%
2 (intermediate)	9	22.5 ± 2.1	≤30%
3 (strong)	21	40.2 ± 2.0	>30%

^1^ Ordinal judgment by 2 experienced investigators. ^2^ Mean ± SD (%) of positive cells. ^3^ In the original publication: 0 (0–5%); 1 (6–33%); 2 (34–66%); 3(>66%).

## Supplementary Materials

The following are available online at https://www.mdpi.com/2077-0383/9/7/2061/s1, Figure S1: Microscopic overview of control and tumor samples, Figure S2: EMT-marker isotype controls, Table S1: Antibodies for routine immunohistochemistry, Table S2: Scoring of biomarkers, Table S3: Antibodies for immunofluorescence multichannel cytometry.
