# Supplementary material for: Slug Is A Surrogate Marker of Epithelial to Mesenchymal Transition (EMT) in Head and Neck Cancer
Source: J Clin Med. 2020 Jun 30;9(7):2061. doi: 10.3390/jcm9072061 (PMC7408865; doi:10.3390/jcm9072061)
Supplement: Supplementary file 1 [file jcm-09-02061-s001.pdf]

Supplementary Materials:

Table S1. Antibodies for routine immunohistochemistry.

Antibody	Catalogue Number	Manufacturer	Dilution	References
Slug *	564614	BD Pharmingen Austria	1:100	[1]
programmed cell death 1 ligand 1 (PD-L1)	13684	Cell Signaling Technologies	1:100	[2]
Ki67	E059	Linaris	prediluted	[3]
Survivin	Ab76424	Abcam	1:100	[4]
matrix metalloproteinase 9 (MMP9)	RP066-05	Diagnostic Biosystems	1:100	[5]
carbonic anhydrase IX (CA-IX)	NB100-417SS	Novus Biologicals	1:100	[6]
CD44	ABIN1020059	Antibodies online	prediluted	[3]
excision repair cross complementation group 1 (ERCC1)	ABIN 197720	Antibodies online	1:100	[7]
p16	6595294001	Roche Ventana	pre-diluted	[8]
p53	5267102001	Roche Ventana	1:1000	[8]
Isotype-control mouse IgG1	760-2014	Roche Ventana	pre-diluted	[9]
Isotype-control rabbit IgG	02-6102	Invitrogen Life Technologies	1:100	[10]
Universal secondary antibody	760-4205	Roche Ventana	prediluted	[8]

* The same anti-Slug antibody was used for brightfield image cytometry

Table S2. Scoring of biomarkers

Biomarker	References	Ordinal Score for Expression			
		**0 (Absent)**	**1 (Weak)**	**2 (Intermediate)**	**3 (High)**
Slug **, Ki-67, Survivin, CD44	[11,12]	≤5%	≤33%	≤66%	>66%
PD-L1	[13]	<1% of all cells		<1%	
CA-IX	[14]	<10%		11-49%	>50%
ERCC1	[7,15]	0%	1-9%	10-49%	≥50%
MMP 9	[16] *	0%	1-9%	10-29%	≥30%
$p16^{INK4}$	[8,17,18]	<60%		≥60%	
p53	[8,19]	no staining ***	scattered positive reaction	accumulated diffuse staining ***	

* The cut-off between score 2 (intermediate) and 3 (strong) was modified by the authors to 30%, in comparison to the original citation were the cut-off was set at 50%. ** For statistical analysis Slug score was dichotomized into negative (Score 0, 1) and positive (Score 2, 3). *** Based on published data of Bouchalova and colleagues [12,19], p53 immunohistochemistry reaction can be considered as (i) "regular pattern", displayed as scattered positive reaction in some tumor cell nuclei (scored: 1–2), which is related to wild-type p53 gene and to (ii) altered, "irregular pattern", which is displayed as either no staining in any tumor cell nuclei (scored: 0) or accumulating diffuse staining tending to be present in all tumor cells nuclei (scored: 3). The irregular pattern is a reliable sign of p53 mutations and/or protein accumulation [12,19].

Table S3. Antibodies for immunofluorescence multichannel cytometry.

**Antibody**	**Fluorochrome on Secondary Antibody**	**Catalogue Number**	**Manufacturer**	**Dilution**	**References**
anti-pan-cytokeratin	Alexa FL 488	760-2595	Roche Ventana	pre-diluted	[20]
E-cadherin	Alexa FL 594	760-4440	Roche Ventana	pre-diluted	[21]
ß-catenin	Alexa FL 488	610153	BD Transduction Laboratories	1:500	[22]
vimentin	Alexa FL 594	M3200	Linaris (Spring Biotech)	1:100	[20]
Isotype-control mouse IgG1		760-2014	Roche Ventana	pre-diluted	[9]
Isotype-control rabbit IgG		02-6102	Invitrogen Life Technologies	1:100	[10]
secondary goat anti rabbit IgG	Alexa FL 594	A-11012	Invitrogen Life Technologies	1:200	[8,20]
secondary goat anti mouse IgG1	Alexa FL 488	A-21121	Invitrogen Life Technologies	1:200	[8,20]

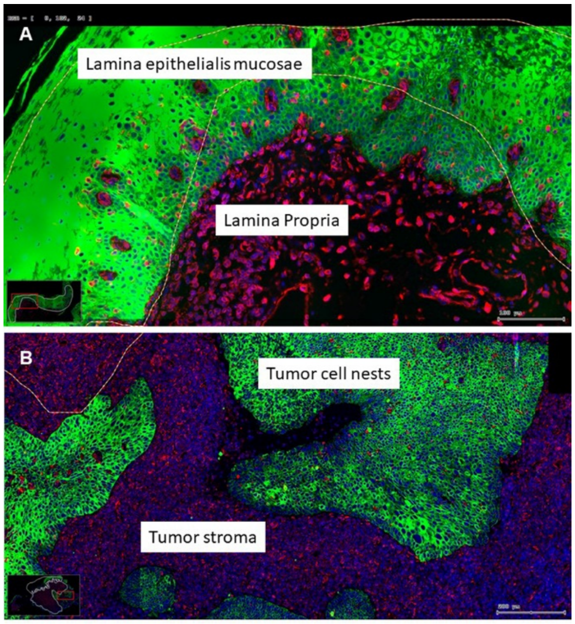

Figure S1. Microscopic overview of control and tumor samples. Immunofluorescence microscopy of **A**: healthy oropharyngeal mucosa and **B**: oropharyngeal cancer with antibodies for cytokeratin (green) and vimentin (red). DAPI was used for nuclear staining Bars: 100 µm. **A**: Control samples were covered by an epithelial layer, consisting of basal cells with larger nuclei (blue) and lower cytokeratin (green) staining at the epithel-stroma interface and several rows of differentiated epithelial cells with higher cytokeratin (green) staining. The epithelial layer was infiltrated by scattered or clustered vimentin cells (red). In the lamina propria mesenchymal cells were present with vimentin reaction and no cytokeratin staining was detected. **B**: Similarly to control samples, the tumor tissue samples were also stained for pan-cytokeratin and vimentin. The tumor cell areas in HNC showed positive cytokeratin reaction (green), and the tumor stroma contained vimentin (red) mesenchymal cells

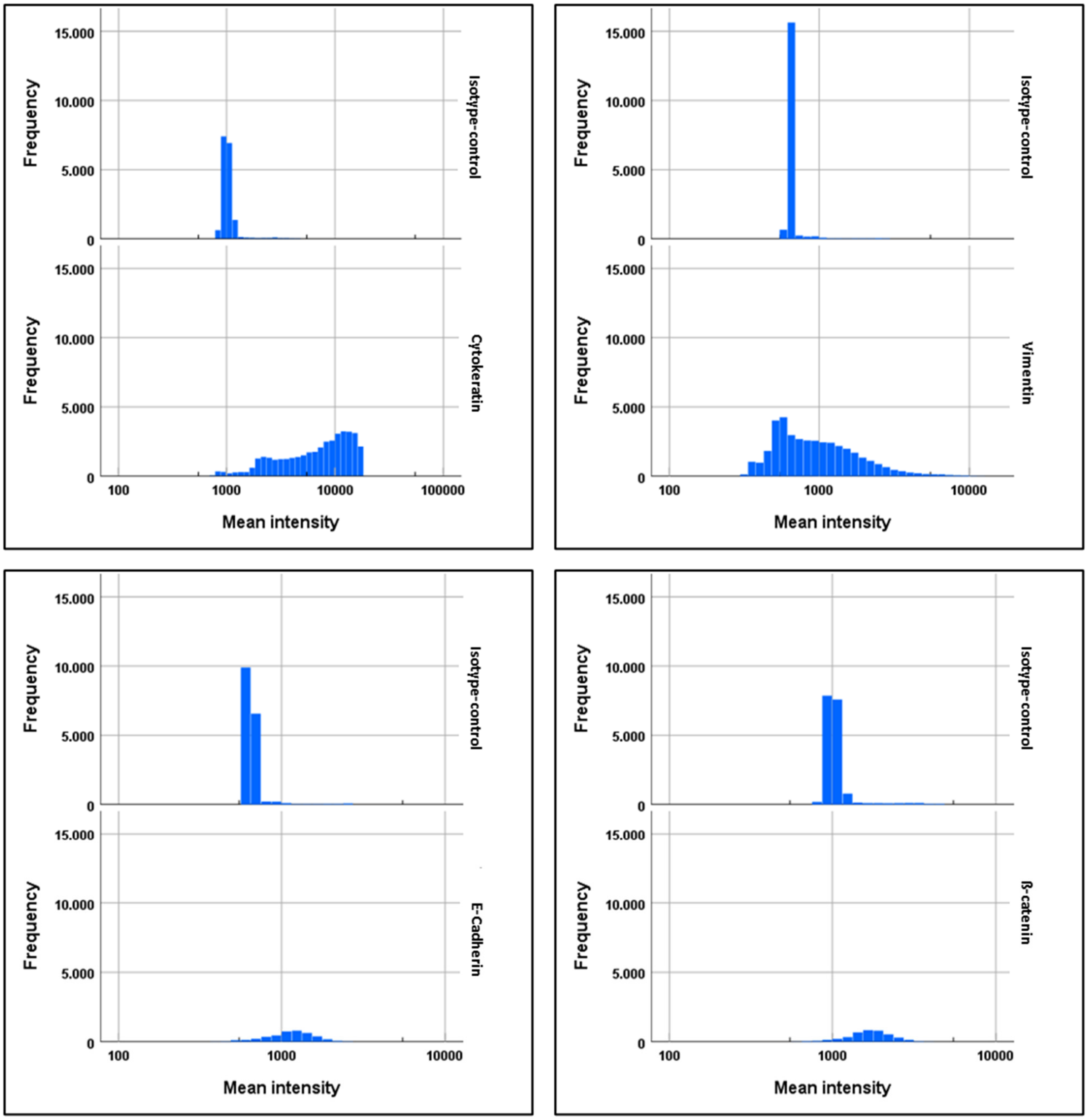

Figure S2. EMT-marker isotype controls.

References

1. Franci, C.; Takkunen, M.; Dave, N.; Alameda, F.; Gomez, S.; Rodriguez, R.; Escrivà, M.; Montserrat-Sentís, B.; Baró, T.; Garrido, M. et al. Expression of Snail protein in tumor-stroma interface. *Oncogene* **2006**, *25*, 5134–5144.
2. Qin, G.; Wang, X.; Ye, S.; Li, Y.; Chen, M.; Wang, S.; Qin, T.; Zhang, C.; Li, Y.; Long, Q. et al. NPM1 upregulates the transcription of PD-L1 and suppresses T cell activity in triple-negative breast cancer. *Nat. Commun.* **2020**, *11*, 1669.
3. Dejaco, D.; Steinbichler, T.; Schartinger, V.H.; Fischer, N.; Anegg, M.; Dudas, J.; Posch, A.; Widmann, G.; Riechelmann, H. Specific growth rates calculated from CTs in patients with head and neck squamous cell carcinoma: A retrospective study performed in Austria. *BMJ Open* **2019**, *9*, doi:10.1158/0008-5472.CAN-06-3822.
4. Steinbichler, T.B.; Alshaimaa, A.; Maria, M.V.; Daniel, D.; Herbert, R.; Jozsef, D.; Ira-Ida, S. Epithelial-mesenchymal crosstalk induces radioresistance in HNSCC cells. *Oncotarget* **2018**, *9*, 3641–3652.

5. Fullar, A.; Kovalszky, I.; Bitsche, M.; Romani, A.; Schartinger, V.H.; Sprinzl, G.M.; Riechelmann, H.; Dudás, J. Tumor cell and carcinoma-associated fibroblast interaction regulates matrix metalloproteinases and their inhibitors in oral squamous cell carcinoma. *Exp. Cell Res.* **2012**, *318*, 1517–1527.
6. Kuthi, L.; Somoracz, A.; Micsik, T.; Jenei, A.; Hajdu, A.; Sejben, I.; Imre, D.; Pósfai, B.; Kóczián, K.; Semjén, D. Clinicopathological Findings on 28 Cases with XP11.2 Renal Cell Carcinoma. *Pathol. Oncol. Res.* **2020**, doi:10.1007/s12253-019-00792-0.
7. Dudas, J.; Schartinger, V.H.; Romani, A.; Schweigl, G.; Kordsmeyer, K.; Marta, P.I.; Url, C.; Kral, F.; Riechelmann, H. Cell cycle association and hypoxia regulation of excision repair cross complementation group 1 protein (ERCC1) in tumor cells of head and neck cancer. *Tumour Biol.* **2014**, *35*, 7807–7819.
8. Dudas, J.; Dietl, W.; Romani, A.; Reinold, S.; Glueckert, R.; Schrott-Fischer, A.; Dejaco, D.; Chacko, L.J.; Tuertscher, R.; Schartinger, V.H. et al. Nerve Growth Factor (NGF)-Receptor Survival Axis in Head and Neck Squamous Cell Carcinoma. *Int. J. Mol. Sci.* **2018**, *19*, 1711.
9. Detrick, B.; Hamilton, R.G.; Folds, J.D. *Manual of Molecular and Clinical Laboratory Immunology*, 7th ed.; ASM Press: Washington, DC, USA, 2006; xxvi, p. 1340.
10. Grzelak, C.A.; Sigglekow, N.D.; Tirnitz-Parker, J.E.; Hamson, E.J.; Warren, A.; Maneck, B.; Chen, J.; Patkunanathan, B.; Boland, J.; Cheng, R. Widespread GLI expression but limited canonical hedgehog signaling restricted to the ductular reaction in human chronic liver disease. *PLloS ONE* **2017**, 12, e0171480.
11. Cappellesso, R.; Marioni, G.; Crescenzi, M.; Giacomelli, L.; Guzzardo, V.; Mussato, A.; The prognostic role of the epithelial-mesenchymal transition markers E-cadherin and Slug in laryngeal squamous cell carcinoma, A.; Martini, A.; Blandamura, S.; Fassina, A. The prognostic role of the epithelial-mesenchymal transition markers E-cadherin and Slug in laryngeal squamous cell carcinoma. *Histopathology* **2015**, *67*, 491–500.
12. Pickhard, A.; Grober, S.; Haug, A.K.; Piontek, G.; Wirth, M.; Strassen, U.; Rudelius, M.; Reiter, R. Survivin and pAkt as potential prognostic markers in squamous cell carcinoma of the head and neck. *Oral Surg. Oral Med. Oral Pathol. Oral Radiol.* **2014**, *117*, 733–742.
13. Ferris, R.L.; Blumenschein, G.; Fayette, J., Jr.; Guigay, J.; Colevas, A.D.; Licitra, L.; Harrington, K.; Kasper, S.; Vokes, E.E.; Even, C. Nivolumab for Recurrent Squamous-Cell Carcinoma of the Head and Neck. *N. Engl. J. Med.* **2016**, *375*, 1856–1867.
14. Perez-Sayans, M.; Suarez-Penaranda, J.M.; Pilar, G.D.; Supuran, C.T.; Pastorekova, S.; Barros-Angueira, F.; Gándara-Rey, J.M.; García-García, A. Expression of CA-IX is associated with advanced stage tumors and poor survival in oral squamous cell carcinoma patients. *J. Oral Pathol. Med.* **2012**, *41*, 667–674.
15. Handra-Luca, A.; Hernandez, J.; Mountzios, G.; Taranchon, E.; Lacau-St-Guily, J.; Soria, J.C.; Fouret, P. Excision repair cross complementation group 1 immunohistochemical expression predicts objective response and cancer-specific survival in patients treated by Cisplatin-based induction chemotherapy for locally advanced head and neck squamous cell carcinoma. *Clin. Cancer Res.* **2007**, *13*, 3855–3859.
16. De Vicente, J.C.; Fernandez-Valle, A.; Vivanco-Allende, B.; Santamarta, T.R.; Lequerica-Fernandez, P.; Hernandez-Vallejo, G.; ALLONCA-CAMPA, E. The prognostic role of claudins-1 and -4 in oral squamous cell carcinoma. *Anticancer Res.* **2015**, *35*, 2949–2959.
17. Kofler, B.; Borena, W.; Manzl, C.; Dudas, J.; Wegscheider, A.S.; Jansen-Durr, P.; Schartinger, V.; Riechelmann H. Sensitivity of tumor surface brushings to detect human papilloma virus DNA in head and neck cancer. *Oral Oncol.* **2017**, *67*, 103–108.
18. Reimers, N.; Kasper, H.U.; Weissenborn, S.J.; Stutzer, H.; Preuss, S.F.; Hoffmann, T.K.; Speel, E.J.M.; Dienes, H.P.; Pfister, H.J.; Guntinas-Lichius, O. et al. Combined analysis of HPV-DNA, p16 and EGFR expression to predict prognosis in oropharyngeal cancer. *Int. J. Cancer* **2007**, *120*, 1731–1738.
19. Bouchalova, P.; Nenutil, R.; Muller, P.; Hrstka, R.; Appleyard, M.V.; Murray, K.; Jordan, L.B.; Purdie, C.A.; Quinlan, P.; Thompson, A.M. Mutant p53 accumulation in human breast cancer is not an intrinsic property or dependent on structural or functional disruption but is regulated by exogenous stress and receptor status. *J. Pathol.* **2014**, *233*, 238–246.
20. Steinbichler, T.B.; Savic, D.; Dejaco, D.; Romani, A.; Kofler, B.; Skvortsova, I.I.; Riechelmann H.; Dudas J. Pleiotropic Effects of Epithelial Mesenchymal Crosstalk on Head and Neck Cancer: EMT and beyond. *Cancer Microenviron.* **2019**, *12*, 67–76.
21. De Re, V. Molecular Features Distinguish Gastric Cancer Subtypes. *Int. J. Mol. Sci.* **2018**, *19*, 3121.

22. Eger, A.; Stockinger, A.; Schaffhauser, B.; Beug, H.; Foisner, R. Epithelial mesenchymal transition by c-Fos estrogen receptor activation involves nuclear translocation of beta-catenin and upregulation of beta-catenin/lymphoid enhancer binding factor-1 transcriptional activity. *J. Cell Biol.* **2000**, *148*, 173–188.
